# Influence of cell–cell distance on the ecology and evolution of microbial communities

**DOI:** 10.1093/ismejo/wrag199

**Published:** 2026-07-26

**Authors:** Ailin Huang, Divvya Ramesh, Yong Nie, Kun Zhao, Miaoxiao Wang

**Affiliations:** Institute of Fundamental and Frontier Sciences, University of Electronic Science and Technology of China, Chengdu, Sichuan 611731, China; School of Life Science and Technology, University of Electronic Science and Technology of China, Chengdu, Sichuan 611731, China; Department of Environmental Microbiology, Eawag - Swiss Federal Institute of Aquatic Science and Technology, 8600 Dübendorf, Switzerland; Institute of Biogeochemistry and Pollutant Dynamics, Department of Environmental Systems Science, 8092 ETH Zürich, Zürich, Switzerland; School of Mechanics and Engineering Science, Peking University, Beijing 100871, China; The Sichuan Provincial Key Laboratory for Human Disease Gene Study and The Institute of Laboratory Medicine, Sichuan Provincial People's Hospital, University of Electronic Science and Technology of China, Chengdu, Sichuan 611731, China; Institute of Fundamental and Frontier Sciences, University of Electronic Science and Technology of China, Chengdu, Sichuan 611731, China; Institute of Fundamental and Frontier Sciences, University of Electronic Science and Technology of China, Chengdu, Sichuan 611731, China; Department of Environmental Microbiology, Eawag - Swiss Federal Institute of Aquatic Science and Technology, 8600 Dübendorf, Switzerland; Institute of Biogeochemistry and Pollutant Dynamics, Department of Environmental Systems Science, 8092 ETH Zürich, Zürich, Switzerland

**Keywords:** cell–cell distance, microbial interactions, spatial ecology, microbial communities, microbial evolution

## Abstract

Microorganisms assemble into spatially structured communities in which neighboring cells are separated by highly heterogeneous cell–cell distances. These spatial distances are not merely geometric features but key ecological variables that determine whether cells can exchange metabolites, compete through toxins, sense one another, or transfer genes, thereby profoundly influencing community assembly, productivity, and the evolution of microorganisms residing in communities. In this review, we synthesize recent progress on how cell–cell distance in microbial communities is determined, as well as how it modulates microbial interactions that lead to diverse ecological and evolutionary consequences. Specifically, we propose several testable conceptual frameworks that help quantify complicated distance–interaction relationships. We discuss emerging imaging, automated analysis, and spatial engineering approaches that now make it possible to quantify and manipulate cell–cell distance with increasing precision. Viewing microbial communities through the lens of cell–cell distance provides a unifying framework for linking microscale spatial organization to microbiome function, offering new opportunities to predict and engineer microbial communities.

## Introduction

Microorganisms colonize virtually every accessible habitat, from the outer surface of the space station to the subsurface miles beneath the land to the intestinal epithelium inside our body. They form intricate multispecies communities, contributing to key ecosystem functions, including Earth’s biogeochemical cycles [1], fertilizing crops [2], purifying wastewater [3], degrading serious pollutants [4], and shaping host-associated microbiomes that influence human health and disease [5]. Although these crucial ecosystem functions can occur in well-mixed or homogeneous environments, many natural and engineered communities are inherently spatially structured, such as soil aggregates, wastewater granular sludge, marine particles, dental plaque, plant root surface, and mammalian mucosal layers. In these communities, different microbial cells occupy different spatial locations, arrange themselves nonrandomly across space, and ultimately develop complicated spatial structures [6].

A key consequence of spatial structure is that it creates varied distances among different microbial cells. The heterogeneities in cell–cell distances profoundly influence the interactions between these cells, which further scale up to drive the assembly and function of the communities, as well as the evolution of microorganisms residing in communities. For instance, spatial proximity between different cells creates opportunities for contact-dependent interactions, driving the emergence of contact-specific cell structures [7]. Cell–cell distance also impacts the contact-independent interactions based on the exchange of the diffusible metabolites. A short cell–cell distance, on the one hand, benefits the metabolic exchange of the essential nutritional metabolites, and thus increases the productivity of the community [8]. In contrast, it also intensifies microbial warfare mediated by biotoxins, potentially destabilizing the community [9].

Despite the recognized importance of cell–cell distances, a central challenge in microbial ecology remains understanding quantitatively how changes in cell–cell separation alter the strength and types of microbial interactions, such as growth rate, fitness, the efficiency of metabolite exchange and signaling, as well as the strength of competition or antagonism [10]. In general, the strength of a single type of interaction typically decays with increasing cell–cell distance. Different interaction mediators, including nutritional metabolites, toxins, signaling molecules, extracellular enzymes, and volatile compounds, possess fundamentally distinct physical and biological properties. Their diffusion, uptake, degradation, leakage, and biological activity thresholds collectively determine how the strength of each interaction decays across space [8, 11]. Because net interaction between microorganisms emerges from the combination of multiple interaction types, microbial interactions can shift dynamically between positive and negative outcomes across different cell–cell distances. However, current studies remain fragmented across interaction types, microbial systems, and spatial scales, limiting the development of a unified conceptual framework linking cell–cell distance to microbial interactions and, ultimately, to the ecology and evolution of microorganisms residing in communities.

Another major challenge is that cell–cell distance simultaneously acts as both a consequence and a driver of microbial interactions. Microbial growth, motility, transitions between solitary and collective lifestyles, fluid flow, and environmental heterogeneity continuously reshape cell–cell distances, which in turn feed back to alter microbial interactions and eco-evolutionary dynamics. This reciprocal relationship generates complex feedback loops between cell–cell distances and microbial interactions that usually remain descriptive but lack predictive frameworks that quantitatively connect cell–cell distance, microbial interactions, and community-level outcomes.

**Figure 1:**
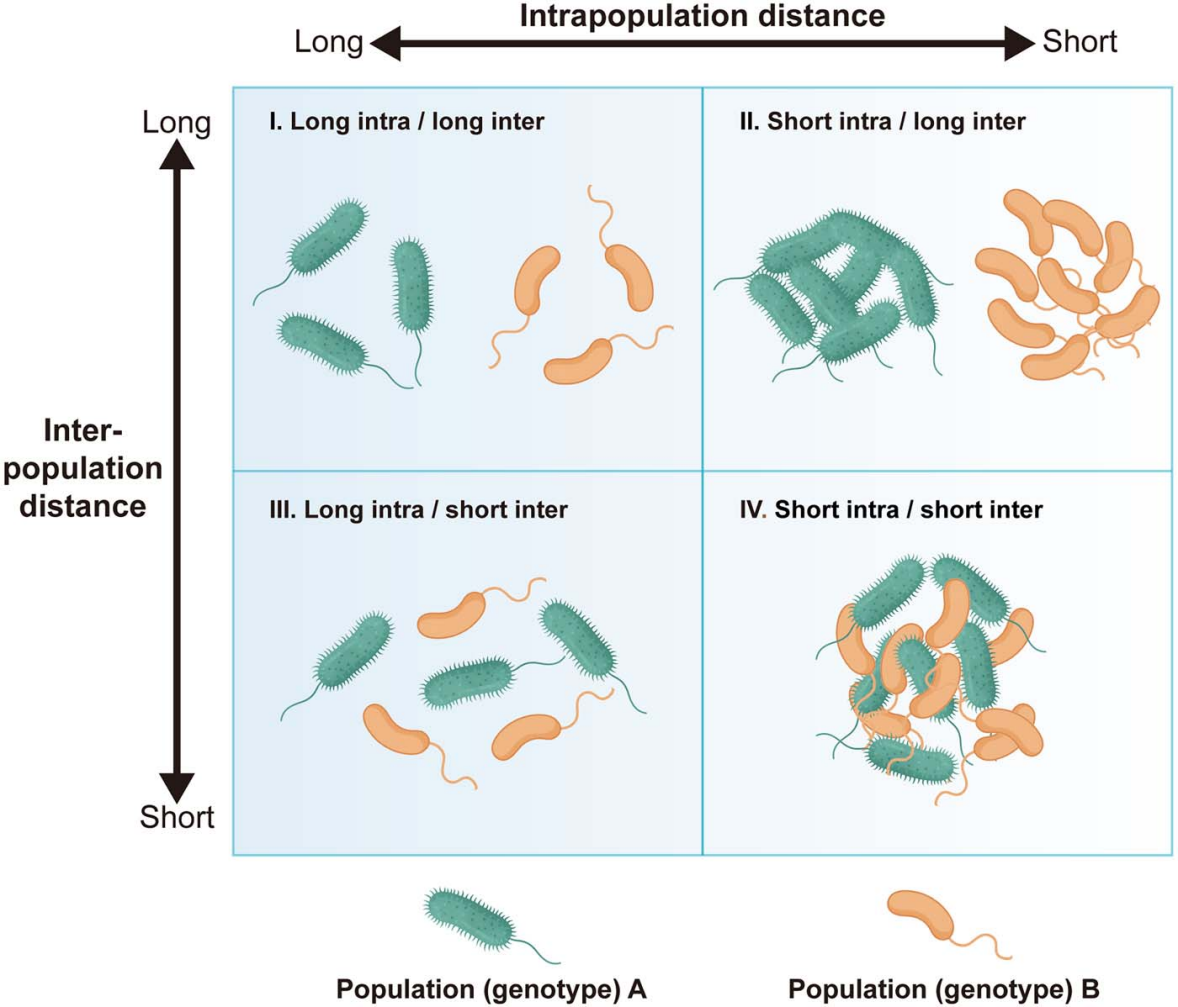
Conceptual overview of the definitions of intra- and interpopulation distances in this review. The four scenarios are arranged in a 2 × 2 grid defined by two distance axes: intrapopulation distance and interpopulation distance. Intrapopulation distance refers to the distance between cells within the same population, and interpopulation distance refers to the distance between cells from different populations. This framework yields four distinct ecological scenarios: (I) long intrapopulation and long interpopulation distances: planktonic, low-density states; (II) short intrapopulation but long interpopulation distances: different populations form discrete, spatially segregated aggregates; (III) long intrapopulation but short interpopulation distances: solitary cells; and (IV) short intrapopulation and short interpopulation distances: dense, intermixed multi-species biofilms.

Here, we summarize a variety of recent studies regarding how cell–cell distances within a community are determined and then govern microbial interactions. We focus on two types of cell–cell distance in this work: (i) the average distance between cells within a population possessing the same genotype (termed intrapopulation distance thereafter); (ii) the average distance between cells from genetically different populations (termed interpopulation distance thereafter) (Fig. 1). We conclude that intrapopulation distance affects both intra- and inter-population interactions, whereas the interpopulation distance mainly governs the interactions among different populations. We propose a series of testable hypotheses to explain the role of cell–cell distance in the ecology and evolution of microorganisms residing in communities, which can be examined through a combination of single-cell-level microscopic analyses with technologies that can rationally position microbial cells in spatial communities. We expect that such pieces of knowledge can advance our understanding of the ecology and evolution of microorganisms residing in communities and provide clues to help manipulate these communities towards desired performance.

## Cell–cell distance governs microbial interactions

### The effects of intrapopulation distance on both intra- and inter-population interactions

Microorganisms from a single population can live a solitary lifestyle, with newly emerging cells rapidly leaving their relatives, exhibiting long intrapopulation distances [12] (Fig. 1: I). Alternatively, they can also form spatially structured biofilms and live a collective lifestyle, in which they live in close proximity to each other with short intrapopulation distances (Fig. 1: II). Such collective lifestyles are common in real spatially structured ecosystems, including microcolonies in root-associated biofilms in the rhizosphere [13], cell clusters in soil aggregates [14, 15], and dense biomass regions in wastewater granules [16–18]. Owing to the differential intrapopulation distances, these two lifestyles have opposite effects on intrapopulation interactions.

Compared with the solitary lifestyle, typically shorter average distances during collective growth generally benefit the intrapopulation interactions mediated by the exchange of public goods (PGs). Typical examples of such PG include nutritional metabolites (e.g. amino acids and vitamins) [19], siderophores [20], and extracellular enzymes secreted for degrading complex compounds [21] or detoxification [22]. Most of these PGs diffuse across the surrounding space to benefit neighboring individuals after they are leaked or actively secreted by the producers. Under solitary lifestyles, in which cells are spatially separated over long distances, these PGs become highly diluted and thus fail to reach potential partners within the population. In contrast, when cells of the same population grow in close proximity, the released PGs accumulate locally, maintaining high concentrations [23]. For example, quantitative models and experiments with *Pseudomonas aeruginosa* demonstrate that short intrapopulation distances in dense cell packing can effectively confine secreted siderophores and enzymes, which maintain high local concentrations of these PGs to support their uptake kinetics [24]. When *Saccharomyces cerevisiae* cells grow as solitary individuals supplied at low sucrose concentrations, the derived exchangeable metabolites, like glucose and fructose, are largely diluted. In contrast, when cells form multicellular aggregates, the locally accumulated glucose and fructose can be shared among neighboring cells [25].

Shorter average distances during collective growth come at a cost: the localized high cell density can easily generate nutrient limitation [26]. For example, computational simulations showed that when multiple cells were in close proximity, the surrounding substrate concentration dropped to ~5% of its initial value [27]. Substantial nutrient consumption establishes strong substrate gradients, resulting in nutrient limitation in the inner biofilm layers, thereby intensifying competition among the cells residing there.

**Figure 2:**
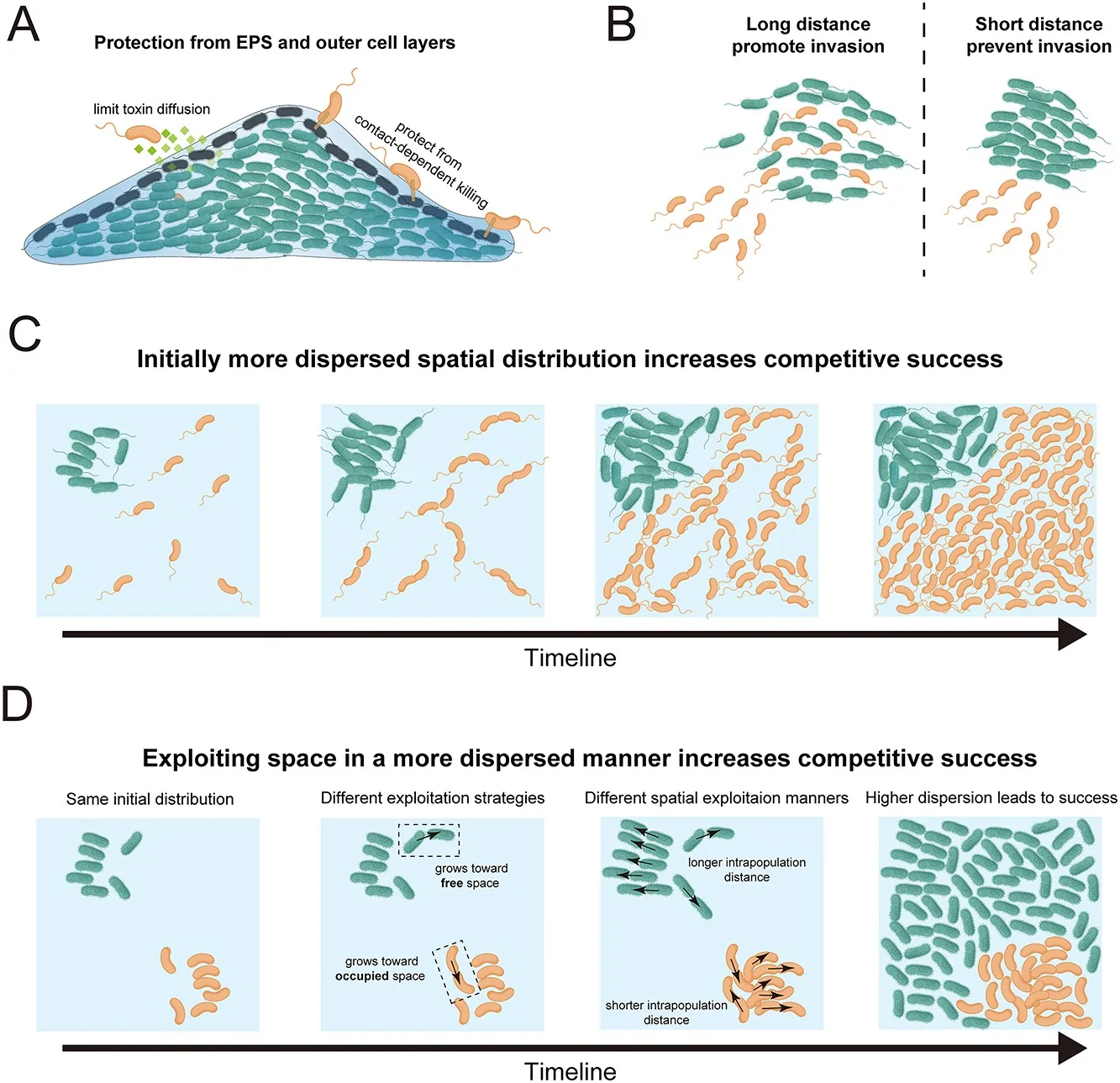
The effects of intrapopulation distance on interpopulation competition. (A) Microbial cells living in dense aggregates or biofilms engage in short intrapopulation distances, in which the EPS and outer cell layers can act as physical barriers to protect interior cells from the attacks of diffusible toxins and contact-dependent antagonists [29, 31, 33, 38]. (B) Tight spatial clustering helps prevent competitors from invading the resident population. Conversely, populations with longer intrapopulation distances are more susceptible to being invaded [33–35, 39]. (C) During spatial competition, populations starting with a more dispersed spatial pattern (longer intrapopulation distance) have higher accessibility to the unoccupied space, ultimately outcompeting populations that initially form tight clusters (shorter intrapopulation distance) [36, 37]. (D) Different spatial exploitation strategies (growing into free versus occupied space) determine the outcomes of spatial competition. A population that actively directs its growth towards free space (maintaining longer intrapopulation distances) exploits resources more efficiently and outcompetes a population that grows towards already occupied space (maintaining shorter intrapopulation distances). Panels (C and D) are adapted from simulation results from our previous work [36].

In addition to the intrapopulation interactions, intrapopulation cell–cell distance also affects the interpopulation competitive interactions. For example, shorter intrapopulation distances lead to a higher local cell density in aggregates or biofilms. This spatial confinement directly leads to the activation of quorum-sensing (QS) pathways, which in turn trigger the massive production of extracellular polymeric substances (EPS) [28]. EPS typically protects the cells inside the aggregates against antagonistic attacks by other populations (Fig. 2A), as extracellular matrices and outer cell layers can act as physical barriers against toxins [29] or contact-dependent antagonism [30, 31]. Beyond matrix-mediated protection, high local cell density can create a nutrient bottleneck through collective nutrient consumption, causing antibiotic-induced death to propagate as a traveling front and protecting interior cells from lethal antibiotic exposure [32]. Furthermore, tight spatial clustering can restrict the invasion by other planktonic populations (Fig. 2B), including resource competitors [33], noncooperators [34], and predators [35].

Spatial proximity among homogeneous cells does not always improve competitive fitness. When different populations compete for new space, more dispersed spatial patterns, where cells maintain larger intrapopulation distances, can instead confer a competitive advantage. Both theoretical models and experiments show that populations with initially more dispersed spatial distributions have a higher probability of occupying more space during subsequent expansion (Fig. 2C), thereby increasing their competitive success [36, 37]. Consistently, agent-based simulations from the same study further showed that a population maintaining the longer intrapopulation distance throughout colonization exhibits the greater competitive advantage [36] (Fig. 2D). These findings indicated that longer intrapopulation cell–cell distances allow populations to exploit space more efficiently and enhance their competitiveness against coexisting populations.

### The effects of interpopulation distance

**Figure 3:**
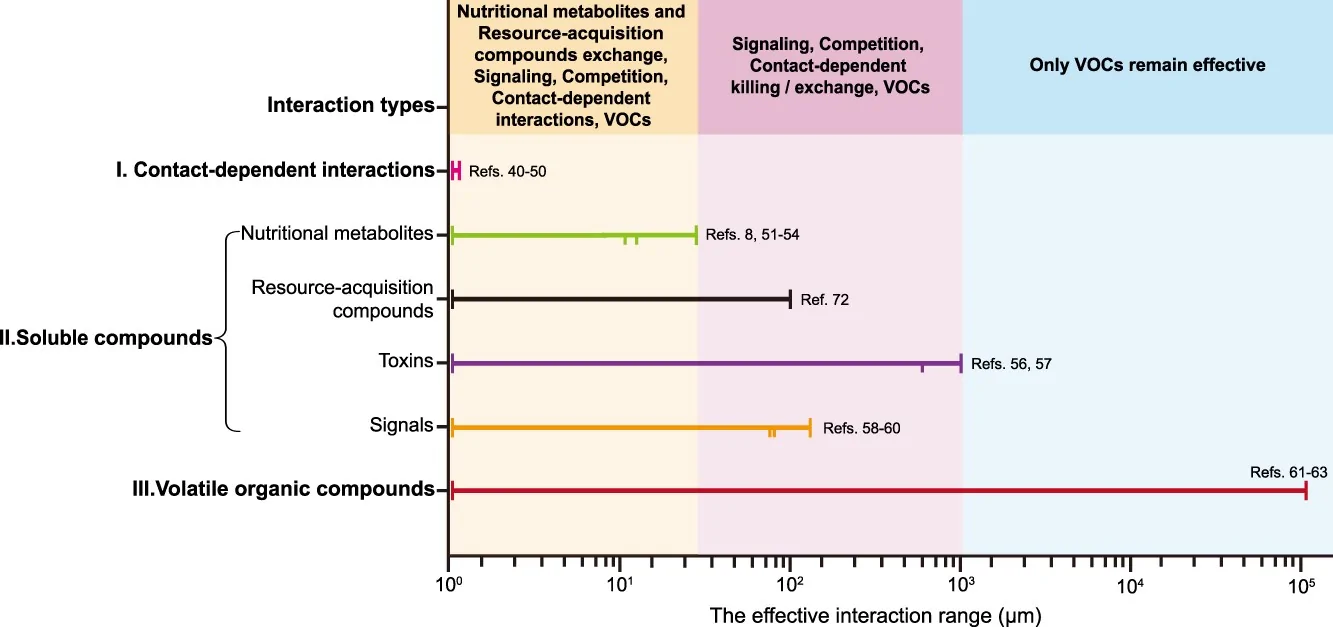
Distance-dependent effects of microbial interaction mediators. Contact-dependent interactions are effective only when cells are in immediate physical proximity. Contact-independent interactions mediated by diffusible soluble or volatile compounds typically weaken as the distance between interacting cells increases. Horizontal bars indicate the approximate effective ranges summarized from the cited studies. Nutritional metabolites, such as amino acids and monosaccharides involved in cross-feeding, generally operate over short distances in the order of tens of micrometers. Resource-acquisition compounds, including siderophores and extracellular enzymes, can mediate interactions over longer distances (up to ~100 μm) because they are not directly consumed and can be reused after releasing nutrients. Toxins often exhibit even larger effective ranges, reaching hundreds of micrometers and exceeding those of most nutritional metabolites (see Box 1 illustration). Soluble signaling molecules typically act over distances of ~100–200 μm. In contrast, VOCs are the primary mediators of long-range interactions over centimeter scales. Reference numbers shown in the figure correspond to those in the main text.

Microbial communities frequently form biofilms comprising a diverse array of microbial genotypes. This highly ordered architecture brings phylogenetically distinct populations into close proximity. The spatial arrangement of these spatially structured communities, particularly the physical distance between interacting genotypes, serves as a decisive factor in determining interpopulation interactions. In particular, different interaction mediators operate across a hierarchical spectrum of effective spatial ranges, spanning from direct contact-dependent interactions to short-range nutritional metabolites, intermediate-range diffusible signals and toxins, and long-range volatile compounds (Fig. 3).

#### Short interpopulation distance enables contact-dependent interpopulation interactions

Short interpopulation distance induces contact-dependent interaction as an effective competitive strategy, which can provide immediate competitive benefits. For example, direct cell–cell contact enables *Streptococcus mutans* to compete against commensal streptococci by inhibiting their ComRS signaling system. This contact-dependent interference directly delivers inhibitory effectors into neighboring cells, effectively decoupling their XIP-ComR feedback loop and quenching their competence-related gene expression [40]. Contact-dependent competition may rely on several molecular nanomachines. One is the contact-dependent growth inhibition mediated by the type Vb secretion system, which can carry a variety of toxin domains that are delivered directly into target bacteria upon cell contact [41]. Another is the Type VI Secretion System (T6SS), which can directly puncture neighboring cells to release toxic effectors into their cytoplasm [42], which is primarily found in many genera of gram-negative bacteria [43–45]. In the gram-positive bacteria, the Esx pathway delivers the LXG toxin into the competitor cells [46]. Spatial proximity creates opportunities for contact-dependent interactions by enabling these molecular nanomachines to execute targeted antagonism through direct cell-to-cell contact.

Short interpopulation distance also promotes contact-dependent metabolic exchange or cross-feeding between neighboring cells. In such interactions, bacteria release essential metabolites, such as amino acids, organic acids (e.g. lactate, acetate, and succinate), and vitamins, which are transported through intercellular channels [47], nanotubes [48, 49], and transient fusion of outer membranes [50]. For instance, *Myxococcus xanthus* employs transient outer membrane fusion mediated by the proteins TraA and TraB to exchange membrane and periplasmic proteins between physically touching cells [50]. Such contact-dependent metabolite exchange can prevent dilution during the diffusion of the metabolites in the extracellular environment.

#### Interpopulation distance determines the strength of the diffusion-based interpopulation interactions

Beyond direct cell–cell contact, microbial interactions can also be mediated by the diffusion of metabolites, toxins, and signals across space. In such cases, cell–cell distance becomes a critical determinant of interaction strength. One related and essential concept is the so-called “interaction range,” defined as the interpopulation distance within which microbial cells can engage in effective interactions through the exchange of metabolites, signals, or other diffusible factors [8]. As the strength of interaction is expected to decay with increasing distance, quantifying the interaction range provides a unifying framework to understand how interpopulation cell–cell distance constrains microbial interactions. Accordingly, we summarize the interaction ranges across different interpopulation interactions and discuss the critical factors that determine these ranges.

Interactions mediated by nutritional metabolites are often effective over a short range. Beyond this effective range, metabolite concentrations frequently drop below the uptake or activation thresholds required by recipient cells, thereby limiting interactions. For example, in the corncob structures of human dental plaque, several taxa are co-aggregated with and thus spatially proximal to *Streptococcus*, which consumes sugars and oxygen and generates metabolites such as lactate, acetate, and CO_2_ that can support their growth [51]. This spatial proximity suggests that diffusion-mediated interactions are strongest at short distances and can shape microscale spatial organization through metabolite gradients. In another example, a synthetic consortium composed of tryptophan- and proline-auxotrophic *Escherichia coli* strains was used to directly quantify their interaction range, which ranged from 3 μm to 12 μm [8]. Similarly, another study found that the interaction range between the phenylalanine and methionine *E. coli* auxotrophs is ~25 μm [52]. These results show that the effective range of amino acids, one common nutritional metabolite [53], is in the range of 3–25 μm (Fig. 3). Similar interaction ranges were reported when *Lactococcus lactis* strains exchanged glucose [54]. In that work, the presence of metabolite-consuming competitors reduced the interaction range to ~15 μm. Several theoretical works demonstrate that the interaction range of nutritional metabolite exchanges typically depends on the leakage, diffusion, uptake, and consumption of metabolites, as well as cell densities [8, 11, 55].

Compared with the effective range of nutritional metabolites, many toxins exhibit a longer effective range compared with the nutritional metabolites (see Box 1 illustration). One model based on Fick’s law suggests that the effective inhibitory radius of antibiotics can reach several hundred micrometers and even approaches ~1000 μm [56]. An aggregate of ~10^5^ cells can secrete antibiotic quantities sufficient to influence the neighboring aggregates located 100 μm away. Complementary experimental work in biofilms shows that toxins with a size of 300–400 Da can penetrate several hundred micrometers into cell clusters within minutes [57].

Box 1 Toxins generally exhibit a larger effective range than nutritional metabolites.

We propose three points to interpret why toxins generally exhibit a larger effective range. First, many nutritional metabolites are not actively secreted but rather leak from the producer cells as metabolic by-products, leading to relatively low production rates [64]. In contrast, toxins are often actively produced and secreted into the extracellular environment, often resulting in their higher yields compared with nutritional metabolites [65]. Second, nutritional metabolites are actively taken up and consumed by neighboring cells, causing a significant decline in concentration, whereas toxins can be removed through target binding [66], cell-entry-associated cleavage [67], defensive enzymatic degradation [68], and intrinsic instability [69]. However, these removal processes are often less continuous than nutritional metabolite uptake, allowing them to exert effects over longer distances [70, 71]. Several experiments and modeling reveal that rapid uptake and consumption by neighboring cells represent the major factor that substantially shortens the interaction range in spatial colonies [8, 11, 55]. Consistent with this opinion, siderophores, secreted by many bacteria for iron scavenging, can mediate interactions within a range ~100 μm, as they are not directly consumed and can be reused after delivering iron to cells [72]. Third, the effective range depends on the biological activity threshold of the molecule. Toxins often act at very low effective concentrations. For example, typical MIC values for many antibiotics fall within the submicromolar to low micromolar range (∼0.1–10 μM) [73]. Therefore, even with a limited diffusion range, toxins are still sufficient to inhibit recipient cells at a distance [74]. In contrast, many nutritional metabolites require higher concentrations to induce measurable growth. For example, *E. coli* auxotrophs exhibit micromolar-scale thresholds for growth, with an arginine auxotroph requiring ~100 μM arginine and a methionine auxotroph requiring ~10 μM methionine [75].

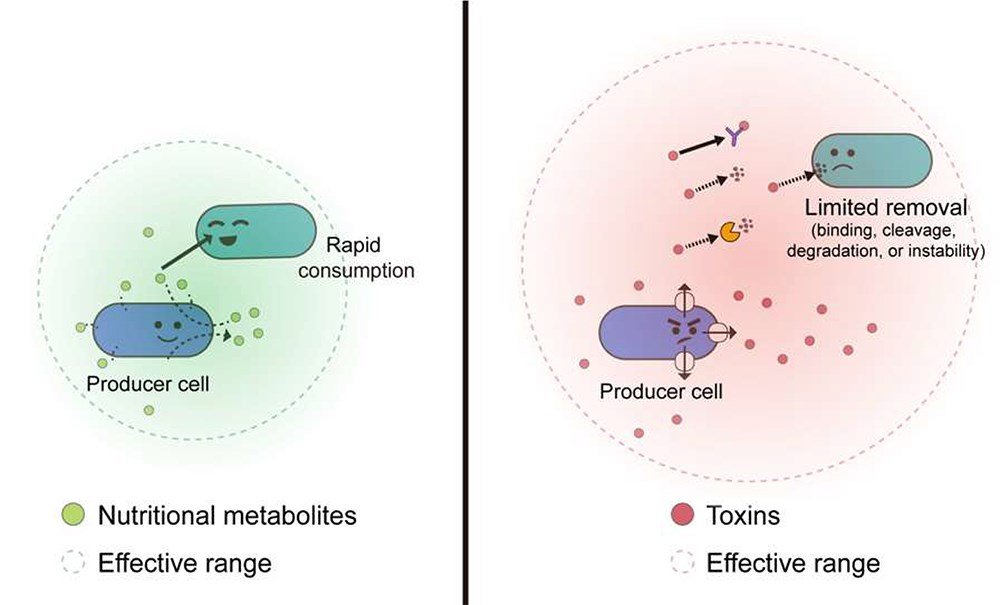

**Box 1 Illustration.** A diagram illustrates why toxins generally exhibit a larger effective range than nutritional metabolites.

The effective interaction range of soluble signaling molecules in microbial communities typically spans tens to a few hundred micrometers and is influenced by production rate, signal stability, and cell densities. A study based on rhizosphere systems demonstrates that signal transmission between individual cells can occur at separations of up to ~78 μm under favorable conditions [58]. In porous monolith microfluidics, acyl-homoserine lactone signaling has been reliably detected across ~75 μm [59]. Moreover, QS signals from aggregates of ≥5000 *P. aeruginosa* cells have been shown to reach neighboring aggregates located 176 μm away, whereas aggregates of ~2000 cells failed to transmit detectable signals to neighboring aggregates [60].

In contrast to soluble compounds, volatile organic compounds (VOCs) enable interactions over centimeter-scale distances, making them well suited for mediating long-range interactions. Functionally, VOCs can mediate long-range antagonism. For example, VOCs released by *Bacillus subtilis* suppress the development of competing biofilms by reducing extracellular matrix gene expression both within and across species [61]. Similarly, *Pseudomonas fluorescens* produces 1-undecene to lethally inhibit *Legionella pneumophila* in physically separated compartments [62]. Beyond antagonistic interactions, VOCs can also serve as long-range beneficial signals. For example, Fungal volatiles function as long-distance communication signals and trigger exploratory growth in *Streptomyces colonies* even under physical separation [63].

## Ecological and evolutionary consequences of the distance-dependent interactions

### Intra-population distance affects the growth of spatial microbial populations

**Figure 4:**
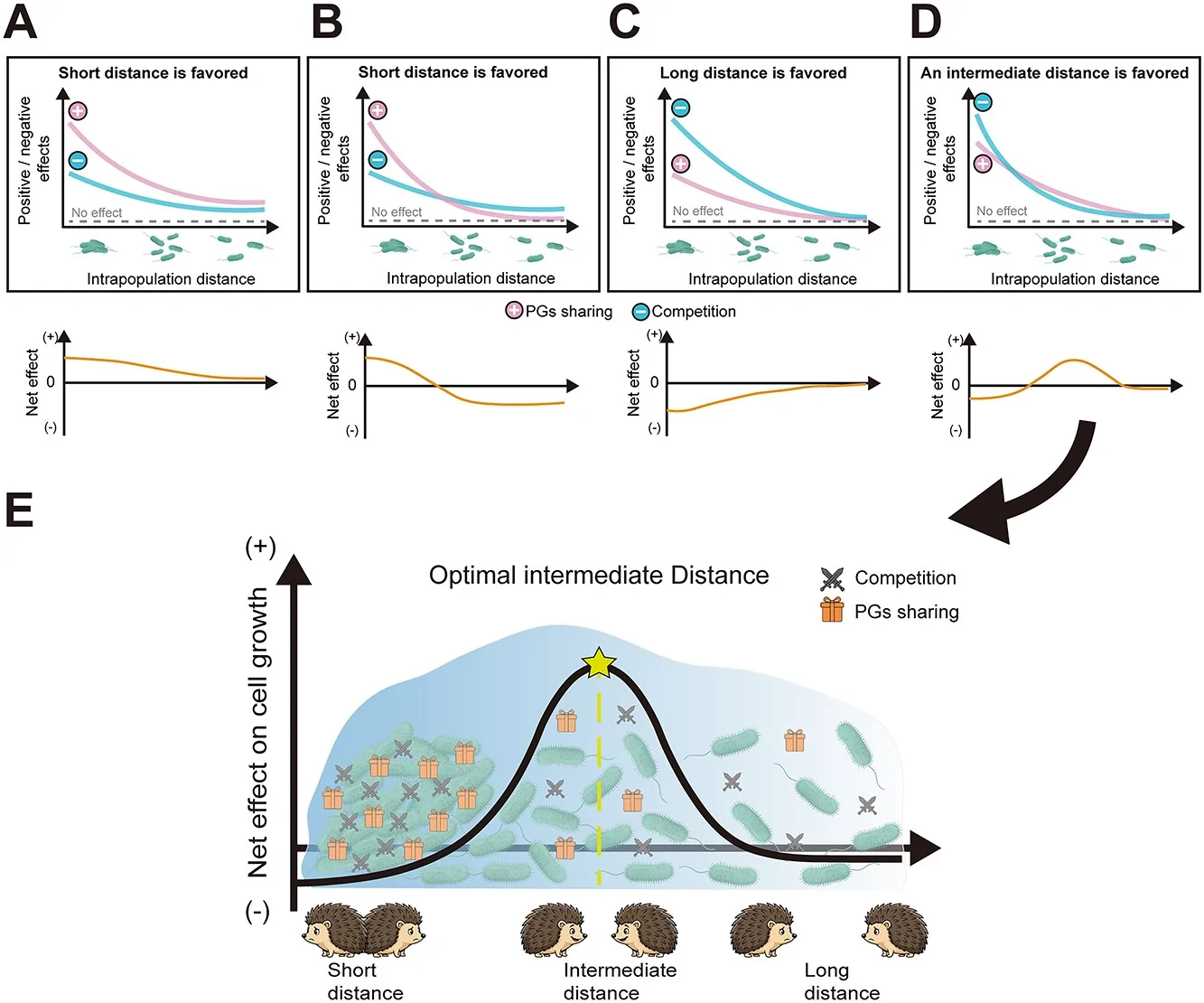
Different scenarios of how intra-population distance affects the growth of spatial microbial populations. (A–D) Conceptual models illustrating the net effect on recipient cell growth over relative intrapopulation interaction ranges. The overall outcome is determined by the quantitative relationship between the positive effects of PGs sharing and the negative effects of resource competition. The bottom plots show the resulting net effect on population growth. (A and B) Two different scenarios in which short intrapopulation distance is favored, because the benefit of PGs sharing outweighs the cost of competition at short range, although the distance-dependence of the PG-sharing benefit differs between the two panels. (C) A scenario in which long intrapopulation distance is favored, because the cost of competition exceeds the benefit of PG sharing across most spatial ranges. (D) A scenario in which an intermediate intrapopulation distance is favored, because competition dominates at short distances, whereas PG-sharing benefit becomes maximal at intermediate distances, producing a peak in the net growth effect. (E) The net effect on cell growth exhibits a unimodal distribution with changes in intrapopulation distance under a certain scenario characterized in (D). At short intrapopulation distances, intense resource competition negates the benefits of PGs sharing, analogous to hedgehogs hurting each other with their spines when huddling too closely. At long distances, cells are too far apart to effectively share PGs, resulting in low collective fitness (analogous to hedgehogs shivering from the cold). The optimal intermediate distance represents the peak of the curve, where microbial populations perfectly balance PGs sharing with competitive resource depletion to maximize collective fitness.

As discussed in section The effects of intrapopulation distance on both intra- and inter-population interactions), intra-population distance exerts dual effects on population performance. On one hand, short cell–cell distances intensify competition for limiting resources, thereby decreasing growth. In contrast, short cell–cell distances can reduce diffusional losses of PGs, increasing cooperative interactions and population growth. Figure 4 conceptualizes this trade-off as a balance between two opposing distance-dependent effects: the benefit of PG sharing and the cost of local resource competition. In this framework, the favored distance is determined by the net effect of these two processes on population growth. Population growth is, therefore, expected to depend on a trade-off between the benefit of PG exchange and the cost of resource competition, in which several scenarios can be predicted. First, when the benefit of PG exchange outweighs the cost of local resource competition, short intercellular distances, that is, living in dense cell aggregation, are optimal (Fig. 4A). Second, when the cost of resource competition dominates the benefit of PG exchange across all spatial ranges, large intercellular distances become optimal (Fig. 4B), favoring a dispersed, planktonic lifestyle that minimizes competitive interference. Finally, if competition dominates at very short distances but PG-mediated cooperation becomes advantageous beyond a certain spatial threshold, population growth is maximized at an intermediate cell–cell distance (Fig. 4C). We refer to this scenario as a “hedgehog scenario,” similar to hedgehogs seeking warmth in winter: unable to benefit from each other when too far apart, yet risking injury when too close (Fig. 4D). Microbial cells may regulate their cell–cell distances to maintain an intermediate separation that balances PG exchange and resource competition.

The scenarios in Fig. 4 focus on the effects of intrapopulation interaction on growth. However, in natural and engineered environments, changing and maintaining a certain intrapopulation distance often carry additional costs on growth, which may change the baseline concepts denoted in Fig. 4. In habitats with advective flow, shortening intrapopulation distance may require active movement, chemotaxis, or surface exploration [76–78] followed by cell–cell and cell–surface attachment mediated by EPS, adhesins, pili, or other matrix components to maintain the distance [79–81]. In weak-flow environments, intrapopulation distance can be passively reduced through clonal growth, because cell division naturally places daughter cells close to their mother cells [82]. To increase intrapopulation distance, cells may need to activate motility and secrete matrix-degrading enzymes to escape from biofilms or aggregates [83]. Because these processes consume energy, they may reduce growth and offset the benefits of distance-dependent interactions. We, therefore, predict that the realized optimal distance will depend on the balance between the growth benefit generated by net intrapopulation interactions and the energetic costs of changing or maintaining that distance. When these costs are high, cells may either remain near their initial spatial configuration or adopt a distance close to, but not identical to, the interaction-determined optimum. Accordingly, future models may extend our simplified conceptual baseline by incorporating environment-specific costs of aggregation and dispersal. Such incorporation may help explain transitions in the life cycle of biofilms, including when planktonic cells are favored to attach and aggregate and when biofilm cells are favored to disperse.

### Interpopulation distance affects the co-existence of different populations and determines the productivity of a spatial microbial community

By exerting influences on their interactions, the distance between different populations alters interpopulation coexistence. On the one hand, a long interpopulation distance diminishes the effectiveness of antagonistic weapons and diffusible biotoxins, as well as reduces the intensity of resource competition. As a result, competitive or antagonistic populations can co-exist beyond the limit set by competitive exclusion [84]. In one relevant study, reducing the physical distance between soil microorganisms intensified competition, resulting in decreased coexistence of different species, whereas increased distances favored poorer competitors, thus increasing coexistence [85]. In contrast, a short inter-population distance promotes the cross-feeding of nutritional metabolites. The process of metabolic cross-feeding has been recognized as crucial for sustaining the coexistence of different populations [19, 86]. Therefore, short inter-population distances can increase the co-existence of metabolically interdependent populations.

Interpopulation distance also emerges as a crucial factor that decisively shapes the productivity (here, we mostly discussed biomass productivity, i.e. growth) of a microbial community. In a relevant study, soil bacteria *Xanthomonas retroflexus* and *Microbacterium oxydans* stayed close to each other during the development of a four-species biofilm, thereby facilitating their own establishment [87]. In turn, this process promoted the separation of the competitive *Stenotrophomonas rhizophila* and *Paenibacillus amylolyticus*, thereby enabling enhanced growth of the whole community. Beyond the dynamic behavioral and protective interactions, short interpopulation distance is a strict prerequisite for sustaining community productivity under extreme energy-limiting conditions. In marine sediments, anaerobic oxidation of methane is highly energy-limited. To overcome this thermodynamic bottleneck, anaerobic methanotrophic archaea cooperate with sulfate-reducing bacteria by forming tightly associated multicellular consortia [88]. By compressing their interpopulation distance, these partner cells can exchange electrons directly via direct interspecies electron transfer [89], intercellular wiring [90], or redox conduction [91]. This close spatial association provides both populations with sufficient energy to support their growth and ultimately drives a critical reaction, oxidation of methane, in the global carbon cycling. Short distances create opportunities for interspecies cell–cell signaling, significantly impacting community productivity. For instance, in a multi-species community, *Actinomyces naeslundii* triggers the expression of arginine biosynthesis genes in *Streptococcus gordonii* upon cell–cell contact, leading to enhanced growth of the entire community when exogenous arginine is limited [92]. In spatially structured communities, antibiotic-resistant strains can offer cross-protection to antibiotic-sensitive strains by actively degrading the antibiotics [93, 94]. Short interpopulation distance benefits this protective effect, therefore increasing community-level resistance to antibiotics [95].

**Figure 5:**
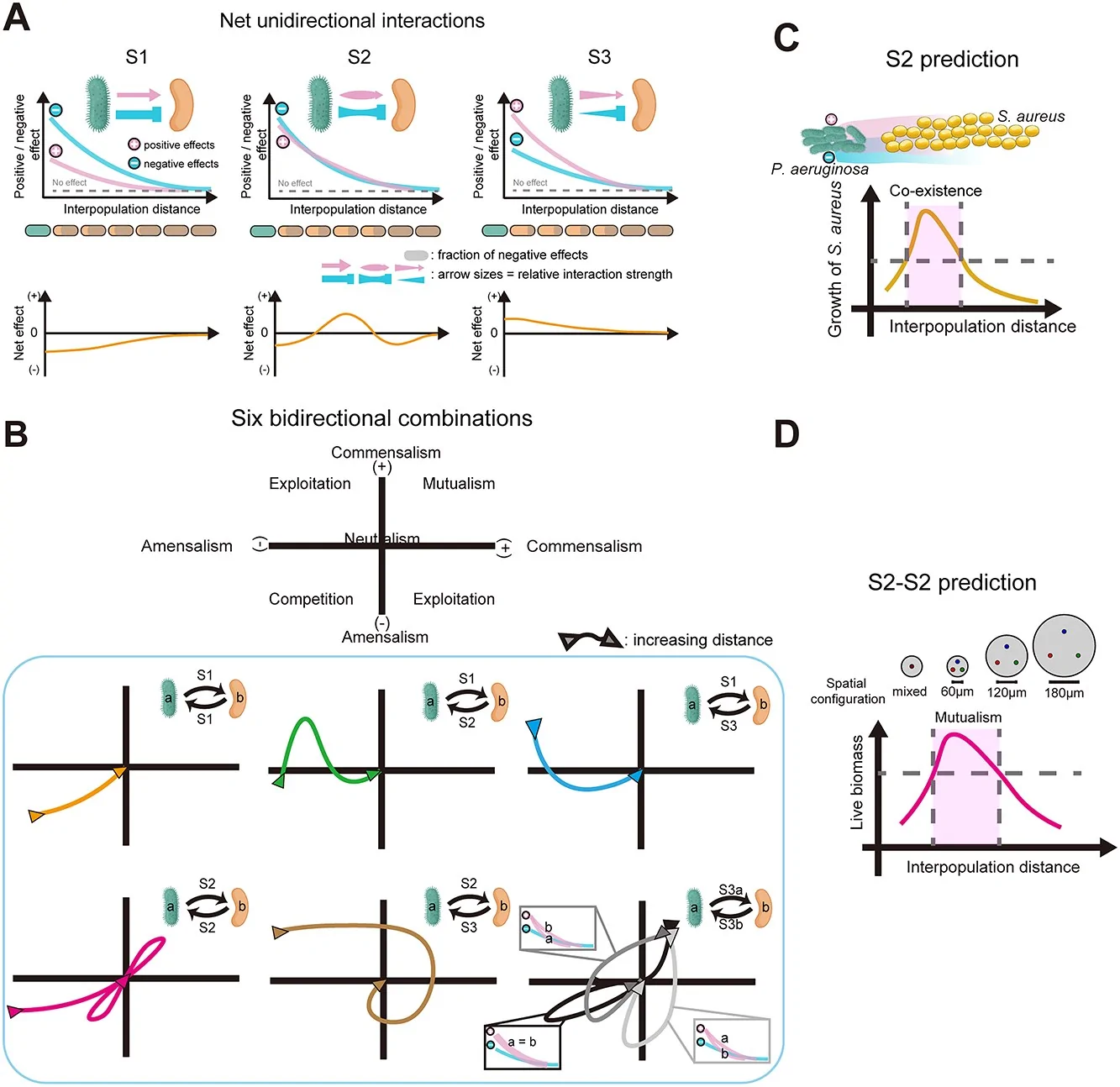
Conceptual models of distance-dependent interpopulation interactions and their ecological outcomes in microbial communities. (A) Three possible profiles characterizing the positive effects of the exchange of nutritional metabolites and the negative effects of toxin-mediated antagonistic interactions on recipient cell growth as a function of relative interpopulation distance. The bottom plots show the resulting net effect on population growth. Here, we assume that toxins act over a longer spatial range than nutritional metabolites (see box 1 illustration). Scenario 1 (S1): toxin-mediated inhibition exceeds the benefits of metabolite exchange across all distances, resulting in consistently negative interactions. Scenario 2 (S2): toxins dominate at short distances, whereas metabolite-mediated benefits prevail at intermediate distances, leading to a positive interaction window flanked by inhibition at both shorter and longer distances. Scenario 3 (S3): positive effects dominate at short distances but are overtaken by negative effects as distance increases. (B) Pairwise combinations of these unidirectional profiles generate distance-dependent trajectories of bidirectional interactions across six distinct combinations. Interaction types include: Mutualism (+, +), Competition (−, −), Exploitation (+, −) or (−, +), Commensalism (+, 0) or (0, +), Amensalism (−, 0) or (0, −), and Neutralism (0, 0). The colored trajectories represent distinct dynamic spatial shifts as interpopulation distance increases, with a triangle marking the starting point and the endpoint. The six panels illustrate these representative trajectories: competition across all distances (S1–S1), competition–exploitation–competition (S1–S2), competition–mutualism–competition (S2–S2), short-range exploitation followed by competition (S1–S3), short-range mutualism followed by competition (S3–S3), and exploitation–mutualism–exploitation–competition (S2–S3). For the scenarios of S1–S1, S2–S2, and S3–S3, only the trajectories when both sides of unidirectional interactions follow the same quantitative relationship with distance are listed. When they follow different relationships, more complex trajectories arise, and we list three possible trajectories when pairing S3 with S3 in the right panel. (C) Consistent with S2, *P. aeruginosa* and *S. aureus* coexist only when their interpopulation distance is within a certain intermediate distance window [96, 97]. (D) Consistent with the bidirectional S2–S2 case, three interacting species stably coexist and achieve substantial growth only at a range of intermediate separation distances.

Within a multi-species community, populations commonly engage in a variety of interaction modes, encompassing both positive and negative effects on the growth of other populations. How the net interaction between two populations changes along interpopulation distance depends on both the interaction ranges and how interaction strength decays with distance. To illustrate this relationship, we consider a simplified yet common scenario in which interactions are mediated by diffusible nutrients (positive effects) and toxins (negative effects). Considering unidirectional interactions from one population to the other, three profiles can arise for how the net interaction effect changes with interpopulation distance (Fig. 5A). In Scenario 1 (S1), toxin-mediated inhibition consistently exceeds the benefits of nutritional metabolite exchange across all spatial scales. Consequently, the net interaction remains permanently negative. Scenario 2 (S2) represents a complex, nonmonotonic dual relationship. Here, toxins dominate at short distances, causing inhibition at short distances. However, metabolite-mediated benefits prevail at intermediate distances, producing a positive interaction window flanked by inhibition at both shorter and longer distances. In Scenario 3 (S3), positive effects dominate at very short distances, shielding the recipient from negative effects at close proximity. But the positive effects are quickly overtaken by inhibitory effects as distance increases, highlighting the dynamic, distance-dependent balance between facilitation and inhibition. Pairing the three unidirectional distance–effect profiles yields six bidirectional combinations. These combinations predict that the pairwise interactions between two microbial populations shift dynamically with their interpopulation distance (Fig. 5B). As different interaction types alter community coexistence and productivity, the distance-dependent interaction patterns described above provide a useful framework for predicting these community outcomes. For example, two recent studies show that *P. aeruginosa* and *Staphylococcus aureus* coexist only when they are spatially segregated at intermediate distances [96, 97]. This outcome arises from dual unidirectional effects exerted by *P. aeruginosa* on *S. aureus*: under tobramycin stress, *P. aeruginosa* both inhibits *S. aureus* through the production of rhamnolipids (negative interaction) and promotes its antibiotic tolerance via the secretion of HQNO (positive interaction). Consequently, the net unidirectional interaction varies with distance and follows our proposed scenario 2, in which positive effects emerge only within a limited intermediate distance window (Fig. 5C), thereby enabling their coexistence [96] and increasing community-level tolerance [97]. When considering pairwise interactions, one study shows that three interacting species stably coexist and achieve substantial growth only at intermediate separation distances, on the order of a few hundred micrometers [98]. Their model shows that this observation is because mutualistic interactions emerge only within a limited intermediate distance window, which is consistent with our interaction regime predicted when both directions follow the unidirectional interaction scenario 2 (Fig. 5D). For simplicity, Fig. 5 conceptualizes interpopulation outcomes as the additive combination of multiple distance-dependent interactions. In real microbial communities, however, different interactions may combine nonadditively. For example, when two populations engage in both negative interactions mediated by inhibitory secretions and positive interactions mediated by PG exchange, the inhibitory compounds may suppress recipient metabolism and consequently alter the uptake or utilization of shared PGs [99]. In such cases, the positive and negative interactions become mechanistically coupled rather than simply additive, potentially shifting the baseline relationship between interpopulation distance and the net interaction outcome predicted in Fig. 5.

### Evolutionary consequences of the distance-dependent interactions

#### Microorganisms evolve distance-related interaction strategies

To adapt to dynamically changing cell–cell distances in nature, many microorganisms evolve both short- and long-range interaction strategies in response to different spatial configurations of the communities. Combining agent-based modeling with competition experiments, one recent study showed that contact-dependent weapons are favored when attacking cells are limited. In contrast, diffusible toxins are more effective when attacking cells are abundant and can collectively overcome dilution, inter-strain mixing, and physical barriers [100]. This finding suggests that interpopulation distance can select distinct competitive machinery. Furthermore, microorganisms also evolve regulatory systems that control the expression of different interaction machinery in response to different cell–cell distances. In *Vibrio fischeri*, the T6SS did not provide a competitive advantage under planktonic conditions, where the interpopulation cell–cell distance is expected to be high [101]. In response, *V. fischeri* evolved to downregulate the expression of T6SS in such conditions. In contrast, the expression of T6SS was activated in response to high viscosity and neutral/acidic pH, conditions commonly encountered in host-specific environments, where cells form biofilms with shorter cell–cell distances. In another similar case, the expression levels of the genes encoding T6SS in *P. aeruginosa* were significantly higher in biofilm cells when compared with those of planktonic cells [102].

#### Cell–cell distance shapes evolutionary opportunities in microbial communities

Interpopulation distance itself can act as a strong selective pressure by determining whether microbial interactions and horizontal gene transfer (HGT) can occur efficiently [103]. Several studies showed that short interpopulation distances driven by metabolic interactions [104], evaporation-induced water flows [105], phage predation [106], and drier conditions [14] promote plasmid conjugation, a common form of HGT [107]. In contrast, long interpopulation distance resulting from population segregation during the range expansion was reported to suppress HGT [108]. Besides plasmid conjugation, other HGT mechanisms are also influenced by spatial organization. For example, the efficiency of phage-mediated HGT (i.e. transduction) depends on the spatial encounter probability among donor, recipient, and phage particles, and is therefore also shaped by interpopulation distance. In one relevant work, two bacterial populations with HGT potential were cultured in a well-defined microfluidic chip, revealing that short interpopulation distances enhance both phage-mediated and conjugation-mediated HGT [109]. Natural transformation, another important HGT mechanism involving uptake of extracellular DNA, may similarly depend on local cell densities and spatial proximity of DNA donors and recipients. Dense spatial aggregates enhance extracellular DNA retention and increase encounter opportunities between donor DNA and recipient cells [110, 111]. These findings demonstrate that short interpopulation distances can facilitate HGT between cells, thereby enhancing gene flow and accelerating community-level genetic adaptation. This spatial dependence is particularly relevant in spatially structured natural communities, such as soil aggregates [14, 15], rhizosphere biofilms [13], as well as activated sludge [112, 113] and microplastic biofilms residing in wastewater treatment plants [18], where high local cell density creates conditions for genetic exchange. For example, recent studies show that microplastic particles in aquatic environments serve as high-density colonizing matrices that mechanically concentrate planktonic cells into short-distance, multi-species biofilms. These microplastic biofilms can enhance cell-to-cell plasmid conjugation frequencies and promote the horizontal transfer of antibiotic resistance genes in aquatic and estuarine environments [16, 17].

#### Cell–cell distance shapes the evolution and stabilization of cooperation

Cell–cell distance affects the evolution of cooperation both within a cooperative population (i.e. homotypic cooperation) and between different populations (i.e. heterotypic cooperation). First, the spatial proximity between different loss-of-function mutants can drive the emergence of cooperation. The Black Queen Hypothesis predicts that mutants that lose energetically costly PG production and rely on ancestral producers are selectively favored. The subsequent emergence of multiple, functionally complementary loss-of-function mutants can further promote the establishment of cooperative interactions [19]. A very recent modeling further suggests that the interaction range of common goods exchange determines whether Black Queen dynamics result in distributed PG production that leads to cooperation, or centralized PG production where one ecotype produces all PGs [114]. The work shows that limited interaction ranges can maintain apparently redundant ecotypes and delay the evolution of cooperation. Several studies also suggest that the successful fixation of loss-of-function mutants critically depends on their spatial proximity to ancestral producers or other complementary mutants [115, 116]. Spatial clustering of functionally complementary mutants promotes the formation of tightly associated clusters, enabling the evolution of cooperation.

Second, short distances within spatial clusters can also localize the accumulation of PGs, and thus limit the exploitation by noncooperators, thereby stabilizing homotypic cooperation [34]. This mechanism has been empirically validated in *S. cerevisiae*, where spatially structured growth allows cooperators to preferentially benefit nearby neighbors and restrict access to distant cheaters [117]. In *Vibrio cholerae*, forming thick biofilms or fluid flow limits the diffusion of liberated nutrients, preferentially retaining benefits within producer clusters and thereby reducing exploitation by cheaters [118].

Third, interpopulation cell–cell distance shapes the stability of heterotypic cooperation. Close proximity between different cooperative populations can strengthen their exchange of PG and exclude noncooperators that remain spatially segregated from cooperative clusters. For example, in a synthetic engineered mutualism of *S. cerevisiae*, spatial self-organization favors cooperative strains and progressively segregating noncooperative mutants [119]. Similarly, in engineered co-cultures of *Acinetobacter baylyi* and *E. coli* that reciprocally exchange essential amino acids, spatial clustering stabilizes mutualistic cross-feeding by excluding noncooperating auxotrophs from exchanged metabolites [120]. Recent simulations report that during community expansion, selfish gene loss drives reductive evolution, causing gene-deficient noncooperators to be progressively segregated from cooperative clusters, ultimately shaping stable interdependent spatial patterns [116]. Consistently, in a synthetic community composed of *Rhodopseudomonas palustris* and *Vibrio natriegens*, spatial self-organization allows a dependent mutant to physically segregate from its independent ancestor, a spatial separation that counterintuitively stabilizes microbial cross-feeding [121]. Furthermore, a recent eco-evolutionary model highlights that the physical interaction range of common goods can shape Black Queen evolution [122]. These findings suggest that maintaining spatial proximity with cooperative partners and spatially separating from noncooperators may represent an evolutionary strategy that promotes the long-term stability of heterotypic cooperation.

## Factors that affect cell–cell distance

### Abiotic factors

Abiotic environmental factors shape microbial spatial organization through three mechanistic categories: constraining physical movement, establishing physicochemical gradients, and actively repositioning cells via fluid flow (Table 1). In environments like soil and sedimentary matrices, the porous architecture dictates microbial cell–cell distance through dual mechanisms: physical compartmentalization and the formation of structural chemical gradients. Physically, the intricate pore network acts as a barrier to dispersal, creating tortuous paths that physically segregate populations into distinct compartments. This compartmentalization often enforces high intrapopulation clustering (shortening intrapopulation distance) in accessible pores and imposes physical limits on interpopulation mixing (increasing interpopulation distance) [123]. Beyond physical barriers, the limited diffusion in these matrices generates steep, abiotic gradients of electron acceptors, and carbon sources. Counter-gradients of oxygen and carbon, dictated by pore size and connectivity, can drive the spatial segregation of aerobes and anaerobes into different micro-niches, increasing the interpopulation distance and compressing intrapopulation distances inside pores [124].

**Table 1:** **Factors affecting microbial cell–cell distances**.

	Factors	Effects on intrapopulation distance	Effects on interpopulation distance	References
**Abiotic factors**	Pore architecture	−: promotes local clustering	+: physical barriers that segregate populations	[123]
	Chemical gradients	−: cells confined to favorable micro-zones	+: spatial segregation of functional groups	[124–126]
	Stochastic flow	+: washes out unattached cells	+: washes out unattached cells	[127]
	Increasing fluid flow	Context-dependent: −: removes planktonic cells. +: induce the attachment of specific species	Context-dependent: species respond to fluid flow differently	[128, 129]
	Evaporation-induced hydrodynamics	−: promotes contact-dependent intrapopulation interactions	−: promotes contact-dependent interpopulation interactions	[105, 130, 131]
**Biotic factors**	Active cell motility/Chemotaxis	Context-dependent: motility can break clusters or create aggregates	Context-dependent: −: actively navigate other populations; increasing spatial intermixing +: actively escape from other populations	[132–139]
	Active attachment	−: attach to relatives	−: attach to beneficial partners	[140]
	Intrapopulation signaling	Context-dependent: −: when signaling induces EPS/adhesion. +: when signaling induces dispersion	–	[142–145]
	Interpopulation signaling	–	Context-dependent:−: when signaling induces aggregation. +: when signaling induces dispersion	[146, 147]
	Antagonistic interactions	–	+: eliminates neighboring competing cells	[31, 148]
	Cooperative metabolic exchange	–	−: recipient cells tightly surround producers to benefit	[149]
	High inoculum size	+: leads to clustering	+: leads to homogeneous clustering	[150, 151]
	Genetic drift at expanding frontiers	–	+: random migration of pioneer cells leads to the spatial segregation of populations	[152, 153]

Note: “−” indicates shortened cell–cell distance, “+” indicates increased cell–cell distance.

Chemical gradients can also be generated within dense microbial aggregates (e.g. biofilms). These gradients effectively restrict specific populations to spatial niches where their physiological requirements are met, thereby increasing the interpopulation distances [125]. A typical example of this gradient-driven organization is observed in aerobic granular sludge used for wastewater treatment. Due to the rapid consumption of oxygen at the granule surface, a strong radial oxygen and nutrient gradient is established, creating oxic outer layers and anoxic cores. This gradient causes aerobic ammonia-oxidizing bacteria to predominantly inhabit the aerobic zone on the surface of granular sludge, whereas anaerobic ammonia-oxidizing bacteria and denitrifying bacteria occupy the anoxic zone within the granular sludge, thereby spatially separating these two groups [126].

Distinct from the structural constraints of pore architecture or diffusion gradients, fluid flow, including hydrodynamic shear and evaporation, serves as dynamic forces that actively reposition cells and exert a dynamic effect on cell–cell distance. In habitats like the phyllosphere or soil, flow is often stochastic and of low viscosity. Events such as rainfall act as periodic reset mechanisms that wash out unattached cells and transiently maximize cell–cell distances before re-colonization occurs [127]. In contrast, the mammalian intestinal tract presents a high-viscosity environment governed by continuous peristalsis. Here, the shear forces can prevent microbial attachment and impose a strong selective pressure that removes planktonic or weakly attached cells, increasing the average cell–cell distance [128]. However, one recent study shows that higher fluid flow counterintuitively increases the attachment of bacteria *S. aureus* and *Enterococcus faecalis*, that is, decreases their intrapopulation cell–cell distance [129]. Because fluid flow has different impacts on the attachment of different bacterial species, it may also change the interpopulation distances when different species co-colonize.

Evaporation-induced hydrodynamics actively reshape interpopulation cell–cell distance. Evaporation typically acts as a concentrating force, modulating cell–cell distances through specific hydrodynamic phenomena such as the Coffee Ring Effect (CRE) and Marangoni Convection. Specifically, the CRE transports cells from the droplet interior to the perimeter, producing a dense annulus of cells at the contact line; alternatively, Marangoni flows can change or partially suppress that redistribution depending on surface tension gradients [105]. As a result, the local cell density significantly increases in the deposition zone and intrapopulation distance substantially reduces, which promotes contact-dependent intrapopulation interactions [130]. This evaporation-driven densification also effectively reduces the interpopulation distance between co-existing species by forcing them into a confined spatial niche, thereby promoting contact-dependent interpopulation interactions that would be rare in dilute conditions [131].

### Biotic factors

Although abiotic forces set the physical stage, biotic factors originating from the microbes themselves and interactions (Table 1) can actively and dynamically modulate cell–cell distances, ultimately refining the spatial architecture of microbial communities.

Active cell motility is one essential behavior that allows bacteria to actively seek out nutrients or escape harmful substances, a process known as chemotaxis [132]. Such directional motility can decrease interpopulation distance by enabling cells to move toward gradients (e.g. nutrients, signaling molecules) created by the other populations. For example, *P. aeruginosa* has been observed to migrate toward secreted factors produced by *S. aureus* [133, 134]. *Pseudomonas aeruginosa* cells were also attracted to the electrical signal released by the *B. subtilis* biofilm [135] and actively navigate towards antibiotics to better attack their competitors [136]. Beyond facilitating exploration of new space, cell motility also influences interpopulation distance by altering the spatial intermixing between different populations. Our recent work demonstrates that motile cells can disperse away from their progenitors after cell division, breaking up clonal clusters, which reduces interpopulation distance to promote metabolic exchange between distinct populations [137]. Similarly, bacterial motility along fungal hyphae decreases interpopulation distances, thereby enabling short-range interspecies interactions [138, 139].

Beyond the dynamic responses mediated by cell motility, active attachment between different species represents another cell behavior for maintaining minimal and stable cell–cell distances. Such attachments may exhibit high specificity, enabling microbes to preferentially associate with kin or beneficial partners and ensure the spatial proximity required for their interactions. For example, obligatory episymbiont Saccharibacteria TM7 can utilize type IV pili-mediated twitching motility to locate and attach to suitable host bacterial surfaces [140].

Although the cell–cell distance influences intrapopulation and interpopulation interactions, these interactions can, in turn, affect the cell–cell distance. Intrapopulation interactions affect intrapopulation distance by regulating transitions between solitary and colonial growth, as well as the porosity of single-species biofilms. A major mechanism underlying such regulation is cell–cell signaling, such as QS and c-di-GMP signaling pathways, which control the production of EPS and surface-active molecules in response to environmental changes [12, 141]. One study shows that *Caulobacter crescentus* forms dense cell clusters primarily in the polysaccharide xylan, yet disperses extensively in the corresponding monosaccharide xylose [142]. Similarly, *Vibrio cyclitrophicus* forms dense cell clusters primarily in the complex polymer, alginate, yet disperses extensively in digested alginate, where nutrients are directly accessible [143, 144]. Cell–cell signaling also regulates the porosity of cell aggregation. In *P. aeruginosa*, disruption of QS signaling (for instance, in *lasR* or *rhlR* mutants) results in less cohesive, highly porous biofilms with increased intercellular distances, whereas QS activation promotes dense, cohesive aggregates with reduced porosity [145].

Interspecific interactions significantly affect both intrapopulation and interpopulation distance. First, signals produced by one species can regulate the transition between solitary and colonial growth in another species, thereby changing its intrapopulation distance. For example, acylated homoserine lactones produced by *Paracoccus* sp. interfere with *Listeria monocytogenes* cell aggregation during biofilm formation [146]. In contrast, autoinducer 2 produced by *Streptococcus oralis* induced the aggregation of *Actinomyces naeslunii* [147].

Second, both cooperative and antagonistic interspecific interactions have impacts on interpopulation distance. Antagonistic interactions, such as T6SS-mediated killing, can eliminate neighboring susceptible competitor cells and thereby reshape interpopulation cell–cell distance [31]. To respond, susceptible competitors may secrete EPS to increase their spatial separation and reduce contact-dependent attack [148]. Conversely, cooperative metabolic exchange can decrease interpopulation distance. Recipient cells clustered around producers benefit from high concentrations of shared metabolites, thereby selectively reducing intercellular distances [149].

Finally, physical and stochastic processes can set and subsequently modify cell–cell distances during colonization and range expansion. Initial inoculum size and whether cells arrive as dispersed individuals or preformed aggregates establish different starting distances [150, 151]. During population expansion from these initial conditions, genetic drift at expanding frontiers can further alter interpopulation distance [152], thereby increasing interpopulation distance over time [153].

## Quantify and manipulate the effects of cell–cell distance

Although previous sections established many conceptual hypotheses for explaining how cell–cell distance governs microbial ecology and evolution, transitioning from these hypotheses to mechanistic proof requires experimental validation. Therefore, it becomes imperative to develop tools for quantitatively measuring and deliberately manipulating these distances within spatial populations and communities. These approaches also provide a direct way to test whether the predicted distance–interaction relationships discussed above are applicable across different microbial systems and spatial environments.

### Quantification of cell–cell distance in the spatial microbial communities

**Figure 6:**
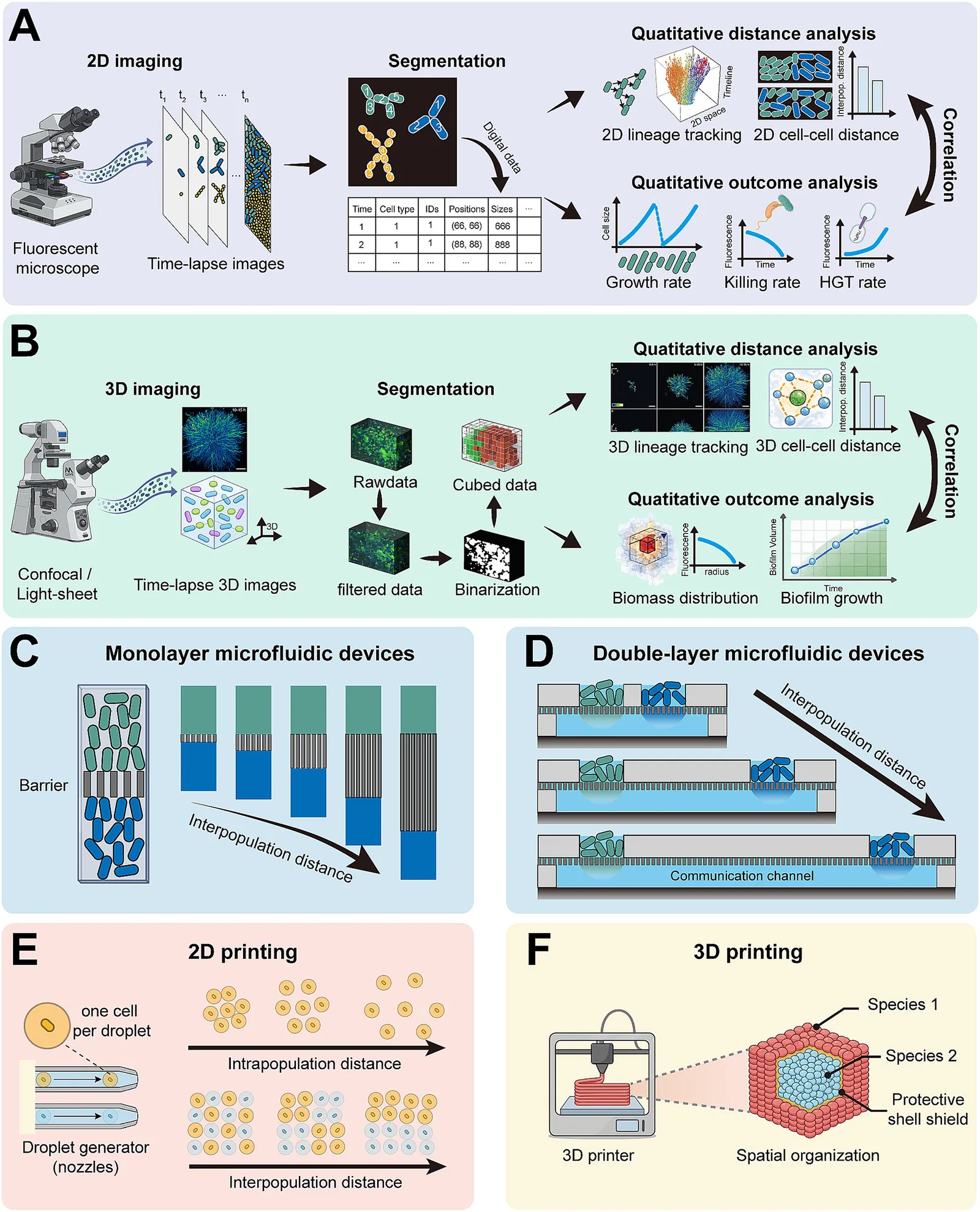
Approaches for quantifying and manipulating cell–cell distances in microbial communities. (A–B) Quantification of cell–cell distances in 2D (A) and 3D (B) systems. (A) Widefield fluorescence microscopy enables the acquisition of 2D time-lapse images, which are subsequently segmented to extract single-cell features, including cell type, size, and spatial position. These data allow quantitative analyses linking distance-related metrics to key ecological outcomes. (B) Confocal or light-sheet microscopy captures high-resolution 3D biofilm structures. Computational tools such as BiofilmQ segment biofilms into discrete volumetric units, enabling quantitative correlations between spatial parameters and community-level functions. (C–F) Manipulation of cell–cell distances using microfluidic devices (C–D), droplet-based approaches (E), and 3D printing (F). (C) Different populations are confined to separate monolayer chambers by porous barriers (e.g. pillars, hydrogels, or membranes), allowing metabolite diffusion without preventing cell mixing and enabling precise control of interpopulation distances [52, 164, 165]. (D) Cells are seeded in separate culture chambers connected by an underlying communication channel, with a nanoporous membrane separating the two layers. This design permits metabolite exchange without disrupting spatial segregation, enabling tunable control of interpopulation distances [98]. (E) Droplet-based systems (e.g. microfluidic generation, electrospray, or bioprinting) produce microdroplets containing different populations that can be deposited into defined 2D arrays, allowing precise control of intra- and interpopulation spacing [167]. (F) 3D printing technologies enable the rational assembly of polymicrobial biofilms with predefined spatial architecture [169].

2D microscopic imaging provides a straightforward means of capturing the spatial distribution of cells within a population or community (Fig. 6A). However, because the volume of image data grows rapidly, the development of automated image analysis (AIA) tools becomes crucial for efficiently extracting quantitative information [154]. When different populations within a community are labeled with distinct fluorescent markers, fluorescence microscopy in a 2D spatial environment, combined with AIA tools, can be used to quantify cell positioning and measure interpopulation distances. However, the number of species that can be simultaneously resolved is limited by the available fluorescent markers and the spectral resolution of microscopes. To enhance the discrimination of different species within a community, many AIA methods have integrated machine learning technologies, enabling accurate recognition of cell morphology for various cell types by leveraging existing datasets [155]. These approaches enable tracking and quantifying the variations in cell–cell distances during community development, and aid in uncovering potential correlations between spatial organization and the lineage history of the population [142, 144]. Additional analyses can be conducted to establish correlations between interpopulation distance and various ecological outcomes, such as contact-dependent killing rate [156], single-cell growth rate [8], and HGT rate [157].

Microorganisms form 3D biofilms. In such cases, confocal microscopy is commonly used to capture the spatial distribution of different cells [158]. It has been widely applied to investigate the spatial development of both single-population and multi-population biofilms (Fig. 6B). In addition to confocal microscopy, light-sheet microscopy has been employed to visualize 3D bacterial biofilms [159]. In comparison to confocal microscopy, light-sheet microscopy presents several advantages, including decreased phototoxicity, rapid imaging speed, and enhanced optical sectioning [160]. These features make it an invaluable tool for investigating cell–cell distances in 3D biofilms. For example, one recent study developed dual-view light-sheet microscopy and found that the positions of founder cells are essential for the development of the *V. cholerae* biofilm [161]. Several AIA tools were developed to capture cell–cell distances in 3D biofilms [155]. One excellent representative is an open-source software named BiofilmQ [162]. BiofilmQ segments 3D biofilms into smaller cubes and extracts various parameters of these cubes, which encompass cell locations and cell–cell distances, enabling the assessment of spatial characteristics in intricate 3D biofilms.

### Rational manipulation of cell–cell distances in spatial microbial communities

In addition to studying cell–cell distance in naturally self-organized spatial structures, recent works also focus on developing methods to engineer desired spatial configurations within microbial communities artificially. One effective approach in this field is to design microfluidic devices that position cells into predefined chambers, thereby achieving desired cell–cell distances [163]. Such a design can be achieved in the microfluidic chambers, which allow the growth of only a monolayer of cells. In these cases, different cell populations are inoculated into separate chambers, and these chambers are separated by specific barriers, such as small pillars [52, 96, 164, 165], monolith [59], agarose gel [166], or nanoporous membranes [98]. These barriers facilitate the diffusion of metabolites but prevent the exchange of cells. In the case where small pillars were used as the barrier, the distances between different populations can be controlled by modulating the width of barriers [52, 164, 165] (Fig. 6C). In other cases, a second layer of channels called the communication channel is introduced beneath the culture chambers, with a nanoporous membrane or agarose gel separating them [98, 166] (Fig. 6D). This communication channel connects the culture chambers, and the interpopulation distances can be modulated by adjusting the distance among the culture chambers.

Bioprinting-based spatial engineering is another major category of cell–cell distance engineering, in which different cell populations are precisely positioned into predefined architectures (Fig. 6E and F). In such set-ups, different cell populations are individually inoculated into separate droplets with diameters ranging from 10 to 1000 μm, which are generated from the microorganism suspensions with proper concentrations. By manipulating the distribution of these droplets, the desired intra- or interpopulation distances can be achieved. For example, one study developed a droplet-printing method that enables the arrangement of different bacterial strains in a 2D plane at micrometer length scales, allowing for the monitoring of their growth and interactions [167]. Using this technology, they created diverse initial spatial patterns using a synthetic consortium composed of competitive populations, with varying interpopulation distances (Fig. 6E). Their work reveals the profound impact of different interpopulation distances on ecological outcomes, including the persistence of specific bacterial genotypes, the ability of strains to shield each other from toxins, and the overall productivity of the community. Recent advancements in 3D printing technology open up new avenues for investigating the impact of cell–cell distance in 3D polymicrobial biofilms [168]. One notable study developed a 3D printing strategy that allowed for the organization of multiple populations of bacteria within different 3D geometries, including adjacent, nested, and free-floating colonies [169]. Using this approach, they printed a 3D biofilm consisting of *S. aureus* and *P. aeruginosa* (Fig. 6F), demonstrating that a small aggregate of *S. aureus* could exhibit significant resistance to β-lactam antibiotics by being enclosed within a protective shell composed of *P. aeruginosa*.

## Concluding remarks

Across spatially structured habitats, variation in intra- and interpopulation distances shapes the strength and types of microbial interactions, thereby influencing species coexistence, as well as community assembly, productivity, and evolution. In this review, we distinguished two types of cell–cell distance: (i) intrapopulation distance, primarily governing interactions among cells of the same genotype and also affecting competition with other populations; (ii) interpopulation distance, mainly determining the interactions between genetically distinct populations. We highlight that cell–cell distance is not merely a passive consequence of spatial growth, but a dynamic ecological variable determined by multiple abiotic and biotic factors, and both emerges from and feeds back on microbial interactions.

A central theme emerging from this review is that the ecological consequences of cell–cell distance are context-dependent. Short intrapopulation distances can promote positive interactions by increasing the local share of nutrients, yet they can also intensify competition for resources. Likewise, short interpopulation distances can facilitate contact-dependent antagonism, metabolic exchange, signaling, and HGT. Increasing interpopulation distances can weaken contact-dependent antagonism and also shift the balance among diffusible mediators with different interaction ranges. Driven by these distinct trade-offs, we explicitly propose a central prediction: microbial populations and communities do not passively default to maximum aggregation or maximum segregation. Instead, collective fitness may peak at intermediate or context-specific spatial configurations, where the benefits of signaling and metabolic exchange are balanced against the costs of resource competition and antagonism. We acknowledge that testing this prediction directly is challenging because spatial organization and microbial interactions often form reciprocal feedback. Overcoming this hurdle requires shifting from purely observational studies to highly controlled, manipulative frameworks. A potential way to combine agent-based modeling with precision spatial positioning of interacting microorganisms, which enable directly track how spatial configurations shape ecology and evolutionary outcomes. Cell–cell distance is itself governed by multiple factors, including abiotic structure, hydrodynamics, chemical gradients, cell motility, adhesion, signaling, interspecific interactions, and stochastic demographic processes. This reciprocal coupling between spatial organization and interaction makes microbial communities highly dynamic and drives their spatial self-organization. Understanding this coupling will be essential for linking microscale spatial structure to macroscale ecosystem function.

Looking forward, the above hypotheses can be directly examined by combining quantitative single-cell imaging, AIA, and experimental platforms that enable precise manipulation of microbial positioning. A mechanistic understanding of how cell–cell distance governs microbial ecology will not only deepen our understanding of natural communities but also provide a practical framework for engineering the spatial structures of microbiomes to improve their stability, productivity, and functions.

## Data Availability

Data sharing is not applicable to this article because no data sets were generated or analyzed.
